# Endovascular Thrombectomy >24-hr From Stroke Symptom Onset

**DOI:** 10.3389/fneur.2018.00501

**Published:** 2018-07-04

**Authors:** Nathan W. Manning, Jason Wenderoth, Khalid Alsahli, Dennis Cordato, Cecilia Cappelen-Smith, Alan McDougall, Alessandro S. Zagami, Andrew Cheung

**Affiliations:** ^1^Department of Interventional Neuroradiology, Institute of Neurological Sciences, Prince of Wales Hospital, Randwick, NSW, Australia; ^2^Department of Interventional Neuroradiology, Liverpool Hospital, Liverpool, NSW, Australia; ^3^Ingham Institute for Applied Medical Research, Sydney, NSW, Australia; ^4^Prince of Wales Clinical School, University of New South Wales, Sydney, NSW, Australia; ^5^Florey Institute of Neuroscience, Parkville, VIC, Australia; ^6^Department of Radiology, Liverpool Hospital, Liverpool, NSW, Australia; ^7^Department of Neurology and Neurophysiology, Liverpool Hospital, Liverpool, NSW, Australia; ^8^South Western Sydney Clinical School, University of New South Wales, Sydney, NSW, Australia; ^9^Department of Neurology, Institute of Neurological Sciences, Prince of Wales Hospital, Randwick, NSW, Australia

**Keywords:** endovascular thrombectomy, ischaemic stroke, penumbra, reperfusion treatment, delayed-window, ASPECTS scores

## Abstract

**Background:** Trials have demonstrated efficacy for endovascular thrombectomy (EVT) for anterior circulation acute ischaemic stroke (AIS) up to 24-h from symptom onset. The magnitude of effect suggests benefit may exist beyond 24-h.

**Objectives:** To perform a retrospective review of all patients undergoing EVT for anterior circulation LVO stroke beyond 24-h from symptom onset and assess safety and efficacy.

**Methods:**A prospectively maintained database of EVT patients treated at two comprehensive stroke centers between January 2016 and December 2017 was retrospectively screened. Patients undergoing EVT for anterior circulation AIS >24-h from symptom onset were selected.

**Results:** A total of 429 AIS patient underwent EVT in the study period. Five patients treated >24-h from symptom onset were identified. The median age was 72 (range 42–84); median ASPECTS 8 (range 6–8); median baseline-NIHSS 9 (range 4–17); and median time from symptom onset to groin puncture 44 h and 55 min (range 25:07-90:10). One patient underwent CT perfusion imaging. The remaining four patients were selected based on non-contrast CT brain and CT-angiography. Two patients had tandem cervical carotid lesions and underwent acute stenting. Modified thrombolysis in cerebral ischaemia (mTICI) 3 reperfusion was achieved in four patients. No hemorrhagic transformation occurred. All patients were alive at 90-day follow-up. Four patients achieved functional independence at 90-days (mRS 0-2).

**Conclusion:** Endovascular thrombectomy for AIS patients beyond 24-h from symptom onset appears to be safe and effective in this limited study. There is a need for further evidence-based trials of benefit vs. risk in very prolonged time windows.

## Introduction

Endovascular thrombectomy (EVT) is an established treatment for acute ischaemic stroke secondary to large vessel occlusion (LVO) of the anterior circulation (1). Recent randomized control trials (RCTs) have demonstrated a marked treatment effect for EVT versus standard care out to 24-h from symptom onset. These RCTs incorporated advanced neuroimaging techniques to select patients with small infarct cores (2, 3). Patients selected in this way presumably represent a subset of stroke patients with slow progression of the ischaemic core into the penumbra (4). In such patients it is possible that the treatment window may extend well-beyond 24-h. As ischaemic changes become increasingly apparent on non-contrast computed tomography (CT) brain scans over time (5), it is possible that this modality may have increased sensitivity for determining the infarct core at delayed time windows.

We present a retrospective review of our experience treating acute ischaemic stroke patients with LVO of the anterior circulation beyond 24-h from symptom onset. The aim of this analysis is to explore the safety and effectiveness of EVT in very extended time windows. We hypothesize that EVT can be safely and effectively performed well-beyond 24 h from symptom onset in a subset of patients.

## Methods

We retrospectively reviewed patients treated at two comprehensive stroke centers, servicing a population in excess of 5 million people and accepting patients from multiple metropolitan and regional hospitals. Patient selection for EVT is performed by the interventional neuroradiologist on-call. To be considered for EVT, patients are required to undergo non-contrast CT brain (CTB) and CT-angiography (CTA) head and neck. Advanced neuroimaging, such as CT-Perfusion (CTP) is preferred, but not required. Selection is predicated on an imaging-clinical mismatch and modified by patient factors, such as premorbid function, comorbidities as well as lesion location and perceived technical difficulty. No upper time limit exists for consideration for treatment.

A prospectively maintained database of all EVT patients treated between January 2016 and December 2017 was retrospectively screened. Patients who underwent groin puncture >24-h from symptom onset were identified. Patient characteristics and clinical information, including baseline NIHSS, imaging data, lesion location and ASPECTS (Alberta Stroke Program Early CT Score) were recorded prospectively. The CTA collateral score was assessed retrospectively as per the scoring system used by Tan et al. (6) Briefly; grade 0 = no collaterals, grade 1 = filling of ≤50% of the occluded territory, grade 2 = >50%, but <100% and grade 3 = 100% of the occluded territory. Outcome data, including operator assessed modified Thrombolysis in Cerebral Ischaemia (mTICI) reperfusion grade, evidence of hemorrhagic transformation and 24-hpost-operative NIHSS was collected. The primary end-point for the procedure was the functional outcome at 90-days as assessed by the modified Rankin Scale (mRS). Good functional outcome were considered mRS 0-2. The study was approved by the local ethics committee at each center.

## Results

From January 2016 to December 2017, 429 AIS patients were treated at the two comprehensive stroke centers. Five patients (1.2%) were identified with symptom onset to groin puncture times >24-h.

Four of the patients were men. The median age was 72 (range 42–84) and median baseline NIHSS 9 (range 4–17). All patients had an occlusion of the first segment of the middle cerebral artery. Two patients had tandem lesions, occlusion of the M1 segment and occlusion of the extracranial ICA requiring stenting. Both were pre-loaded with aspirin (600 mg) and clopidogrel (600 mg). Thrombectomy was performed with the combination of local aspiration via an intermediate catheter and simultaneous use of a stent-retriever in all patients, the so-called SOLUMBRA technique. No patients received intravenous thrombolysis. All patients underwent general anesthesia for EVT (Table 1).

**Table 1:** Baseline characteristics.

**Outcome variable**	**Outcome (*n* = 5)**
Gender	male: 4
Median age (range)	72 (42–84)
Median NIHSS (range)	9 (4–17)
Median ASPECTS (range)	8 (6–8)
Median Collateral Score (range)	2 (2–3)
M1, MCA, *n*	5
Tandem cervical ICA, *n*	2
IV-tPA, *n*	0
General Anesthesia, *n*	5

*NIHSS, National Institutes of Health Stroke Scale; ASPECTS, Alberta Stroke Program Early CT score; M1, MCA, first segment of the middle cerebral artery; ICA, internal carotid artery; IV-tPA, intravenous tissue plaminogen activator*.

All five patients were transferred from their presenting hospital to the treating comprehensive stroke center (CSC) for the purposes of EVT. The median time from INR (interventional neuroradiology) contact to arrival at the treating CSC was 2 h and 45 min (range 00:00–04:10). All five patients had known stroke onset time. The median time from symptom onset to qualifying imaging was 43 h (range 17:21–88:30). The median time from symptom onset to groin puncture was 44 h and 55 min (range 25:07–90:10) and from imaging to groin puncture was 4 h and 22 min (range 01:40–07:46). The median time from arrival at the treating CSC to groin puncture was 27 min (range 00:11–02:20). The median procedure time was 31 min (range 27–75 min). The median time from stroke onset to reperfusion was 45 h and 45 min (range 25:34–90:41). See Table 2.

**Table 2:** Time metrics.

**Outcome variable**	**Median (hrs:mins)**	**Range (hrs:mins)**
Onset to Imaging	43:00	17:21–88:30
Onset to groin puncture	44:55	25:07–90:10
Imaging to groin puncture	04:22	01:40–07:46
CSC arrival to groin puncture	00:27	00:11–02:20
Onset to reperfusion	45:45	25:34–90:41
Procedure time	00:31	00:27–00:75
INR contact to CSC arrival	02:35	00:00–04:10

*CSC, Comprehensive stroke center; INR, Interventional neuroradiologist on call*.

All patients underwent non-contrast CTB and CTA head and neck at their origin hospital with a median ASPECTS score of 8 (range 6–8). This was the basis of selection for EVT in four patients. The fifth patient underwent repeat imaging, including CTP, on arrival to the treating comprehensive stroke center which demonstrated a persistent large mismatch. Modified thrombolysis in cerebral ischaemia (mTICI) grade 3 reperfusion was achieved in four patients and mTICI 2a reperfusion in the fifth. The mean change in NIHSS at 24-h postoperatively was −4 (range −8 to 0). One patient demonstrated asymptomatic, small volume patechial haemorrhagic trasformation (ECASS 1). No symptomatic intracranial hemorrhage occurred. No imaging or clinical evidence of malignant cerebral oedema was identified. All patients were alive at 90-day follow-up and functional independence (mRS 0-2) was achieved in four patients (Table 3).

**Table 3:** Outcome Data for EVT >24 h.

**Outcome variable**	**Outcome**
mTICI 3, *n*	4
Mean ΔNIHSS at 24 h	−4
SICH, *n*	0
90-day mortality, *n*	0
90-day mRS 0-2, *n*	4

*mTICI, modified Thrombolysis In Cerebral Ischaemia; NIHSS, National Institute of Health Stroke Scale; SICH, symptomatic intracerebral hemorrhage; mRS, modified Rankin Score*.

## Discussion

We present here our experience performing EVT on anterior circulation, LVO AIS patients beyond 24-h from symptom onset. Although these data are limited, they suggest that good functional outcomes may be safely achieved in selected patients treated at this late stage.

Initial analysis suggested an upper limit of ~7 h for treatment effect for EVT in anterior circulation LVO stroke (7). However, more recently, two trials utilizing advanced neuroimaging to select for patients with small ischaemic cores, have demonstrated a large treatment effect out to 24-h from symptom onset (2, 3). Presumably, the use of advanced neuroimaging in the late time window trials selected patients with a relatively stable ischaemic core to penumbra ratio. Previous studies have identified patients with stable ischaemic cores using serial diffusion-weighted imaging (DWI) in the setting of persistent LVO (8). Analysis of the DEFUSE 2 data identified a slow progression phenotype in approximately ~50% of patients with middle cerebral, or internal carotid, artery occlusions (9). Such patients may preserve their ischaemic core to penumbra ratio for a significant time, explaining the benefit of late reperfusion. However, the majority of these patients will fail to permanently stabilize the penumbra and the core eventually expands. This can take days rather than hours (10).

Ischaemic core stability over time may also explain the utility of non-contrast CTB based selection in the current study. Non-contrast CTB has only modest sensitivity for identifying the ischaemic core in early time windows. However, the sensitivity of CT for infarction improves over time as cellular water content increases (5). If the ischaemic core remains relatively stable in size, the ability of CT to accurately detect that core will increase significantly in very delayed time windows.

Regardless of the selection criteria, neuroimaging assesses the ischaemic core and penumbra at a single point in time. It is not possible to predict how long an individual patient may maintain their core to penumbra ratio before completing their infarct. Therefore, reperfusion therapy should proceed in the shortest time frame possible from qualifying imaging. All the patients in the current study were transferred to CSCs for EVT after their qualifying imaging. Therefore, this delay ranged from 1 h and 40 min to as much as 7 h and 46 min. In spite of this, 80% of the patients achieved functional independence at 90 days. Again, this suggests this patient population has a relatively stable core to penumbra ratio.

In the current study CTB determination of ischaemic core appears comparable to CTP in delayed time windows. However, the numbers are too small to draw conclusions. It is likely that CTP will offer greater negative predictive power than CTB. This is an area of ongoing investigation.

The current study is limited by the small number of patients fulfilling the inclusion criteria, the retrospective nature of the analysis and the absence of a control arm. Therefore, the data should be interpreted cautiously. The possibility that these patients may have stabilized, or even improved, without reperfusion cannot be excluded. However, all but one patient demonstrated some neurological improvement with a reduction in the 24-h post-operative NIHSS. This recovery implies restoration of neurological function following reperfusion. Of note, the patient with an unchanged NIHSS failed to achieved successful reperfusion (mTICI 2a). Time to recanalization has previously been demonstrated to be predictive of the risk of hemorrhagic transformation in patients treated with intravenous thrombolysis (11). However, no effect of time on symptomatic intracerebral hemorrhage has been seen in a meta-analysis of the EVT trials (7). We identified no symptomatic intracranial hemorrhage in our limited series, even in patients treated with dual-antiplatelet agents for acute carotid stenting. Recently, high rates of successful reperfusion have been shown to have a larger effect on functional outcomes than time to treatment in early time window studies (12). It is likely that successful reperfusion will be of even greater significance in very extended time windows.

## Conclusion

This small dataset suggests that EVT may be performed safely with low rates of patient morbidity and mortality in appropriately selected patients beyond 24-h from symptom onset. These preliminary data suggest a need for an ongoing registry, or even randomized trial, of EVT beyond 24-h from symptom onset.

## Author contributions

NM conceived of the study in discussion with AC. Data was collected by NM, AC, JW, and KA. Data analysis performed by NM, AC, and DC. Manuscript drafted by NM and edited by all authors.

### Conflict of interest statement

NM is a consultant to Microvention, JW and AC are consultants for Microvention, Stryker and Medtronic. The remaining authors declare that the research was conducted in the absence of any commercial or financial relationships that could be construed as a potential conflict of interest.
